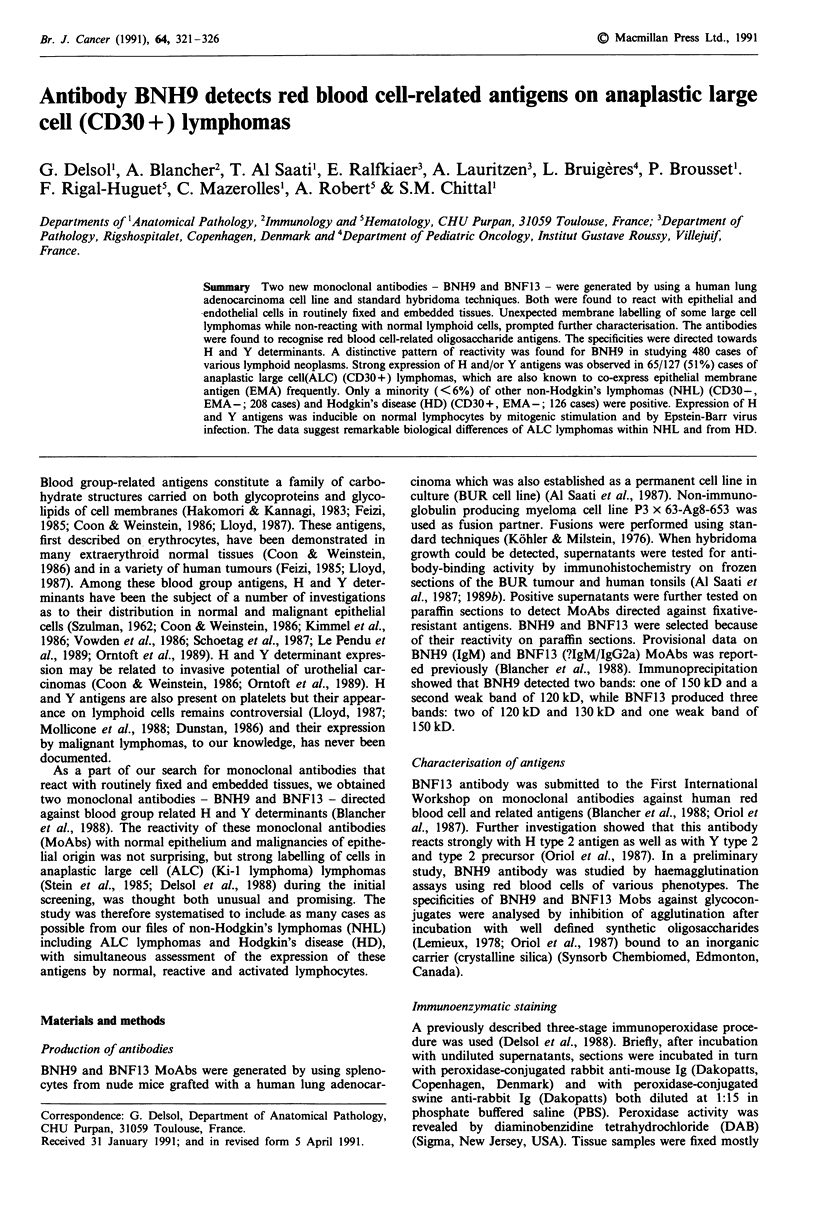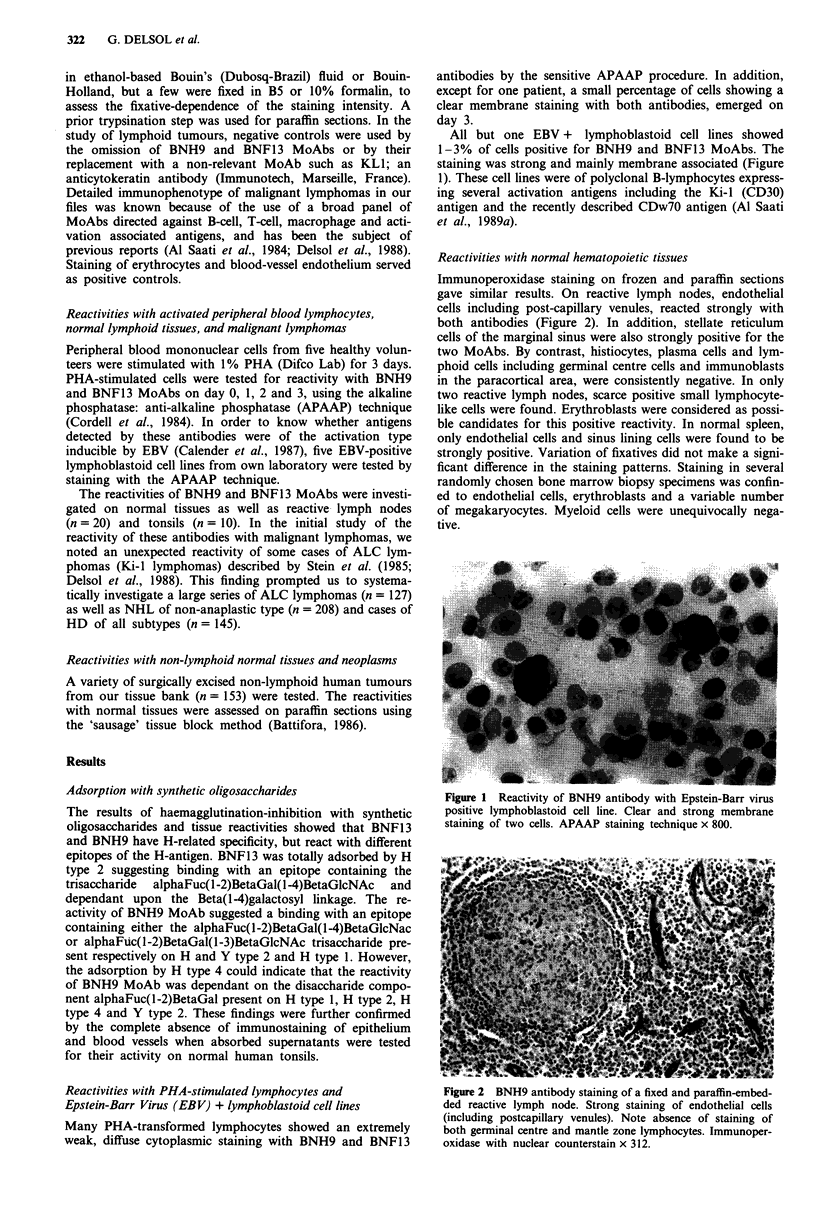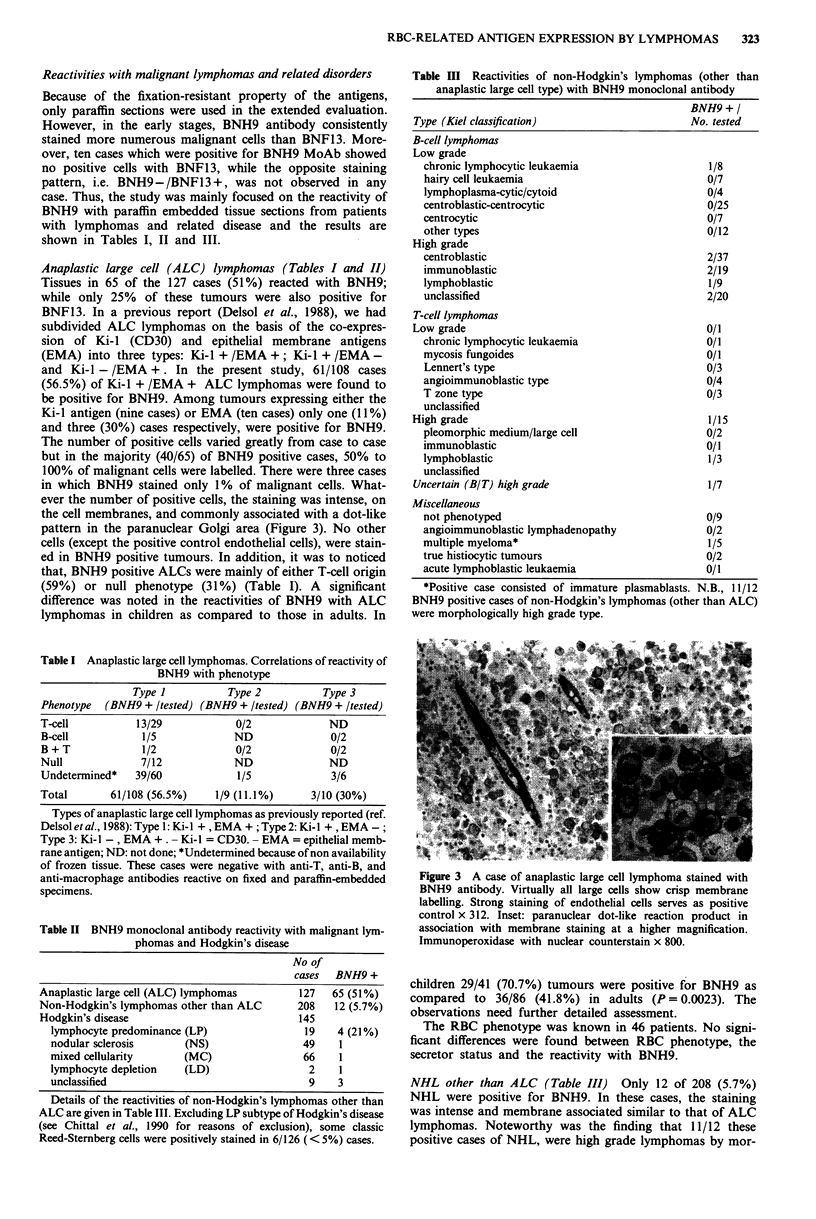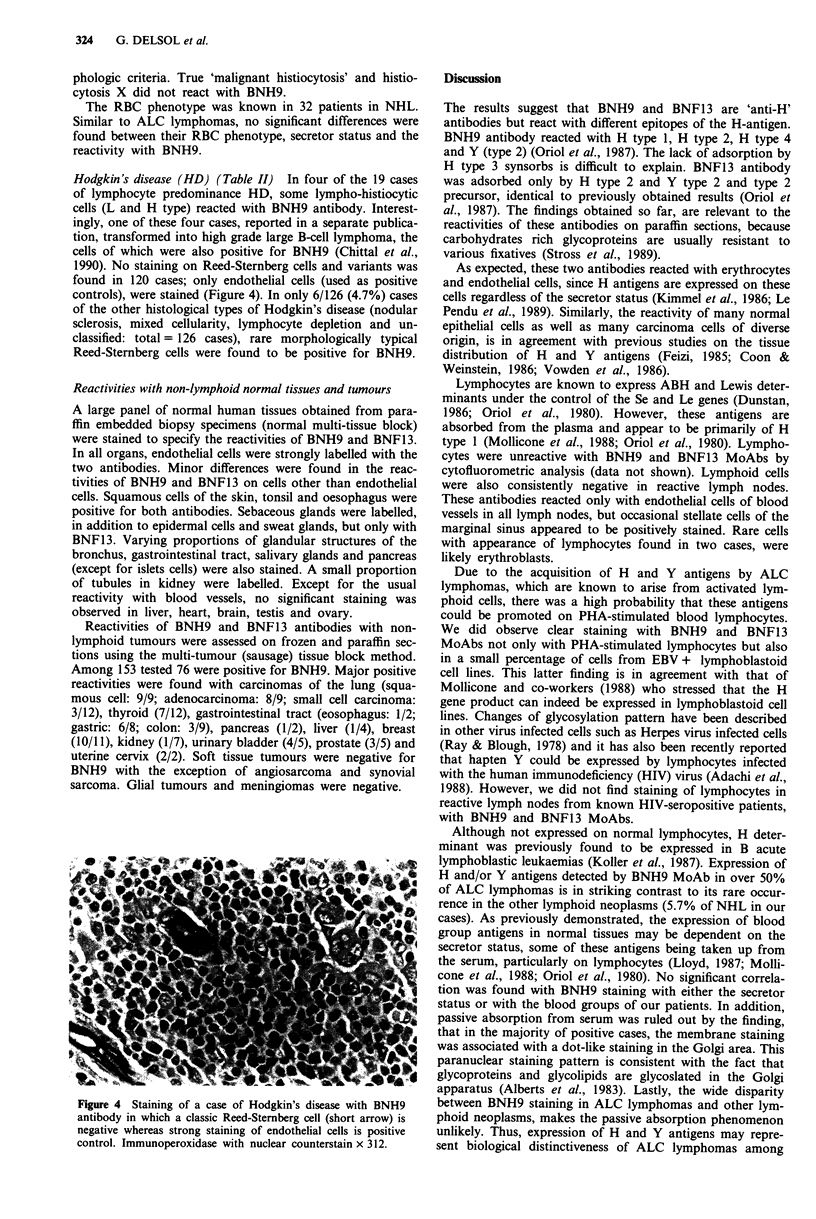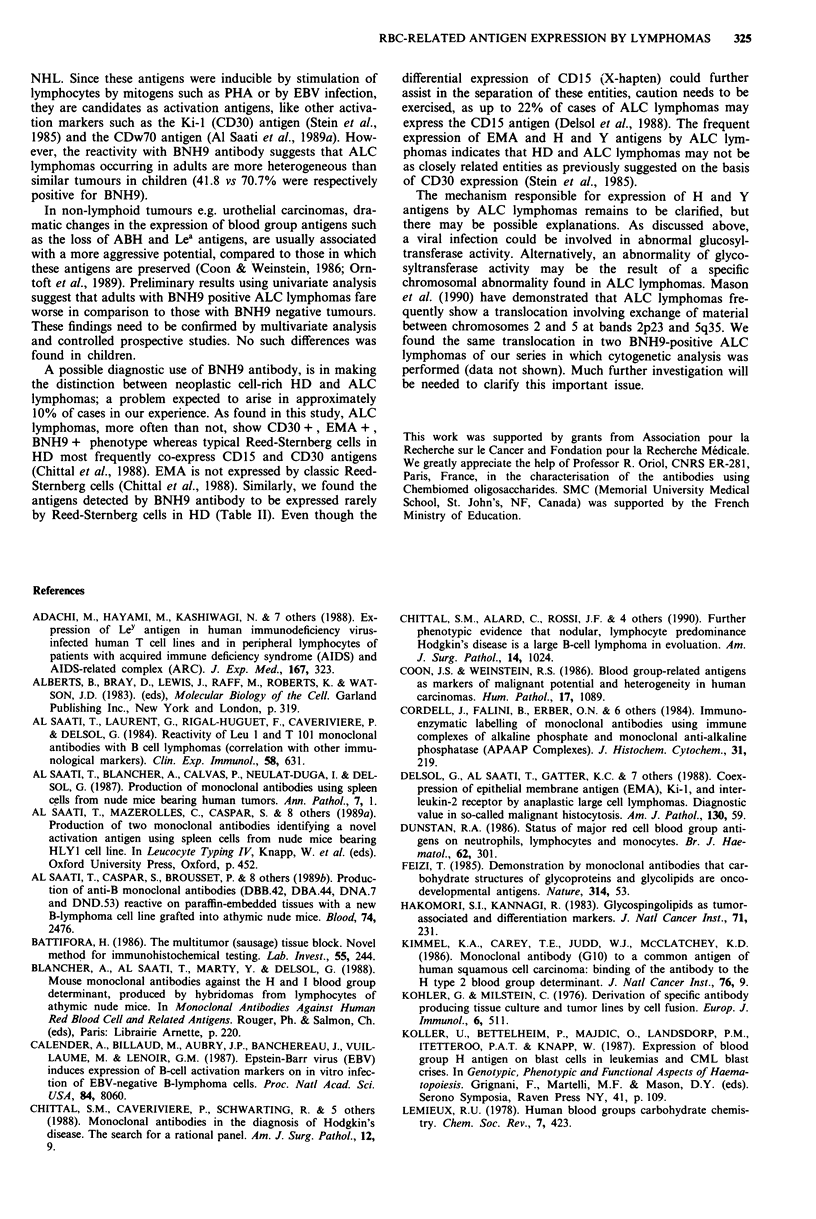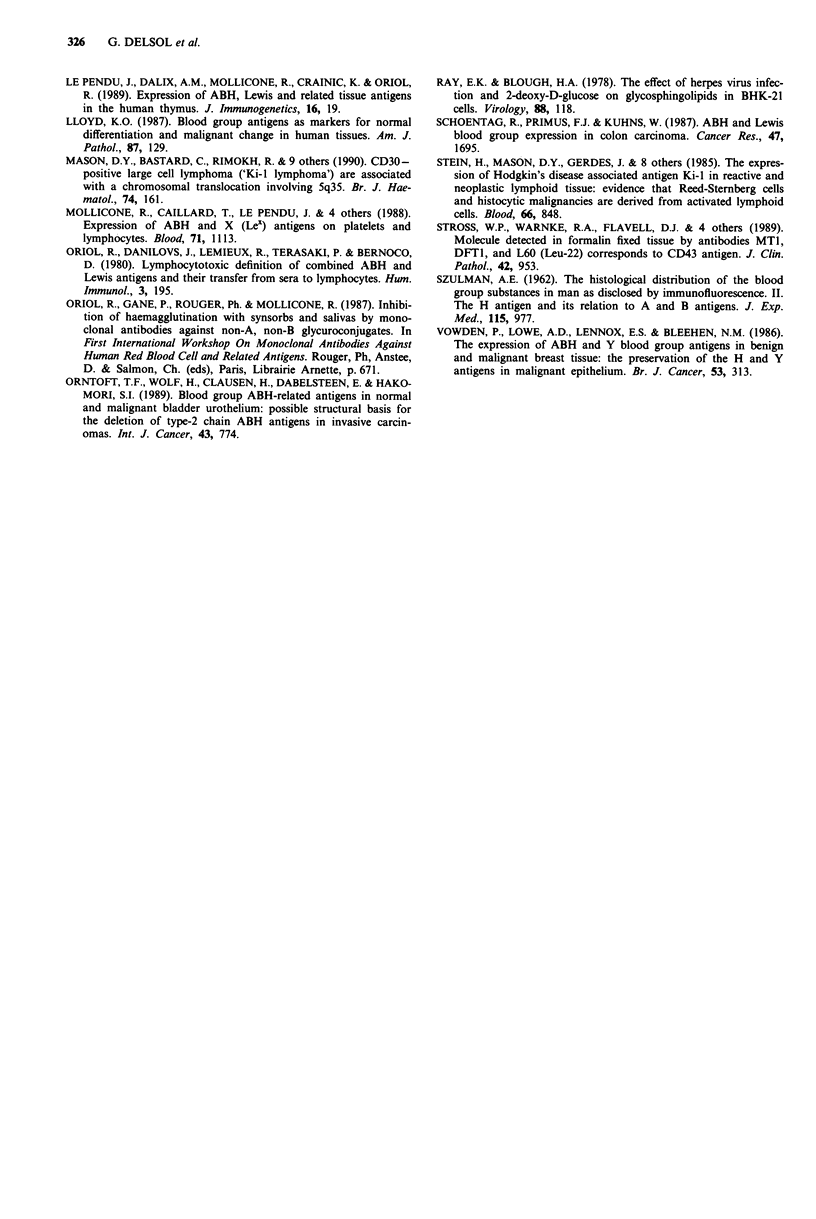# Antibody BNH9 detects red blood cell-related antigens on anaplastic large cell (CD30+) lymphomas.

**DOI:** 10.1038/bjc.1991.299

**Published:** 1991-08

**Authors:** G. Delsol, A. Blancher, T. al Saati, E. Ralfkiaer, A. Lauritzen, L. Bruigères, P. Brousset, F. Rigal-Huguet, C. Mazerolles, A. Robert

**Affiliations:** Department of Anatomical Pathology, CHU Purpan, Toulouse, France.

## Abstract

**Images:**


*Br. J. Cancer* (1991), **64**, 321–326 © Macmillan Press Ltd., 1991

### Antibody BNH9 detects red blood cell-related antigens on anaplastic large cell (CD30+) lymphomas

G. Delsol[1], A. Blancher[2], T. Al Saati[1], E. Ralfkiaer[3], A. Lauritzen[3], L. Bruigères[4], P. Brousset[1]. F. Rigal-Huguet[5], C. Mazerolles[1], A. Robert[5] & S.M. Chittal[1]

*Departments of [1]Anatomical Pathology, [2]Immunology and [5]Hematology, CHU Purpan, 31059 Toulouse, France; [3]Department of Pathology, Rigshospitalet, Copenhagen, Denmark and [4]Department of Pediatric Oncology, Institut Gustave Roussy, Villejuif, France.*

**Summary** Two new monoclonal antibodies – BNH9 and BNF13 – were generated by using a human lung adenocarcinoma cell line and standard hybridoma techniques. Both were found to react with epithelial and endothelial cells in routinely fixed and embedded tissues. Unexpected membrane labelling of some large cell lymphomas while non-reacting with normal lymphoid cells, prompted further characterisation. The antibodies were found to recognise red blood cell-related oligosaccharide antigens. The specificities were directed towards H and Y determinants. A distinctive pattern of reactivity was found for BNH9 in studying 480 cases of various lymphoid neoplasms. Strong expression of H and/or Y antigens was observed in 65/127 (51%) cases of anaplastic large cell(ALC) (CD30+) lymphomas, which are also known to co-express epithelial membrane antigen (EMA) frequently. Only a minority (<6%) of other non-Hodgkin's lymphomas (NHL) (CD30–, EMA–; 208 cases) and Hodgkin's disease (HD) (CD30+, EMA–; 126 cases) were positive. Expression of H and Y antigens was inducible on normal lymphocytes by mitogenic stimulation and by Epstein-Barr virus infection. The data suggest remarkable biological differences of ALC lymphomas within NHL and from HD.

Blood group-related antigens constitute a family of carbohydrate structures carried on both glycoproteins and glycolipids of cell membranes (Hakomori & Kannagi, 1983; Feizi, 1985; Coon & Weinstein, 1986; Lloyd, 1987). These antigens, first described on erythrocytes, have been demonstrated in many extraerythroid normal tissues (Coon & Weinstein, 1986) and in a variety of human tumours (Feizi, 1985; Lloyd, 1987). Among these blood group antigens, H and Y determinants have been the subject of a number of investigations as to their distribution in normal and malignant epithelial cells (Szulman, 1962; Coon & Weinstein, 1986; Kimmel *et al.*, 1986; Vowden *et al.*, 1986; Schoetag *et al.*, 1987; Le Pendu *et al.*, 1989; Orntoft *et al.*, 1989). H and Y determinant expression may be related to invasive potential of urothelial carcinomas (Coon & Weinstein, 1986; Orntoft *et al.*, 1989). H and Y antigens are also present on platelets but their appearance on lymphoid cells remains controversial (Lloyd, 1987; Mollicone *et al.*, 1988; Dunstan, 1986) and their expression by malignant lymphomas, to our knowledge, has never been documented.

As a part of our search for monoclonal antibodies that react with routinely fixed and embedded tissues, we obtained two monoclonal antibodies – BNH9 and BNF13 – directed against blood group related H and Y determinants (Blancher *et al.*, 1988). The reactivity of these monoclonal antibodies (MoAbs) with normal epithelium and malignancies of epithelial origin was not surprising, but strong labelling of cells in anaplastic large cell (ALC) (Ki-1 lymphoma) lymphomas (Stein *et al.*, 1985; Delsol *et al.*, 1988) during the initial screening, was thought both unusual and promising. The study was therefore systematised to include as many cases as possible from our files of non-Hodgkin's lymphomas (NHL) including ALC lymphomas and Hodgkin's disease (HD), with simultaneous assessment of the expression of these antigens by normal, reactive and activated lymphocytes.

### Materials and methods

### *Production of antibodies*

BNH9 and BNF13 MoAbs were generated by using splenocytes from nude mice grafted with a human lung adenocarcinoma which was also established as a permanent cell line in culture (BUR cell line) (Al Saati *et al.*, 1987). Non-immunoglobulin producing myeloma cell line P3 × 63-Ag8-653 was used as fusion partner. Fusions were performed using standard techniques (Köhler & Milstein, 1976). When hybridoma growth could be detected, supernatants were tested for antibody-binding activity by immunohistochemistry on frozen sections of the BUR tumour and human tonsils (Al Saati *et al.*, 1987; 1989*b*). Positive supernatants were further tested on paraffin sections to detect MoAbs directed against fixative-resistant antigens. BNH9 and BNF13 were selected because of their reactivity on paraffin sections. Provisional data on BNH9 (IgM) and BNF13 (?IgM/IgG2a) MoAbs was reported previously (Blancher *et al.*, 1988). Immunoprecipitation showed that BNH9 detected two bands: one of 150 kD and a second weak band of 120 kD, while BNF13 produced three bands: two of 120 kD and 130 kD and one weak band of 150 kD.

### *Characterisation of antigens*

BNF13 antibody was submitted to the First International Workshop on monoclonal antibodies against human red blood cell and related antigens (Blancher *et al.*, 1988; Oriol *et al.*, 1987). Further investigation showed that this antibody reacts strongly with H type 2 antigen as well as with Y type 2 and type 2 precursor (Oriol *et al.*, 1987). In a preliminary study, BNH9 antibody was studied by haemagglutination assays using red blood cells of various phenotypes. The specificities of BNH9 and BNF13 Mobs against glycoconjugates were analysed by inhibition of agglutination after incubation with well defined synthetic oligosaccharides (Lemieux, 1978; Oriol *et al.*, 1987) bound to an inorganic carrier (crystalline silica) (Synsorb Chembiomed, Edmonton, Canada).

### *Immunoenzymatic staining*

A previously described three-stage immunoperoxidase procedure was used (Delsol *et al.*, 1988). Briefly, after incubation with undiluted supernatants, sections were incubated in turn with peroxidase-conjugated rabbit anti-mouse Ig (Dakopatts, Copenhagen, Denmark) and with peroxidase-conjugated swine anti-rabbit Ig (Dakopatts) both diluted at 1:15 in phosphate buffered saline (PBS). Peroxidase activity was revealed by diaminobenzidine tetrahydrochloride (DAB) (Sigma, New Jersey, USA). Tissue samples were fixed mostly

Correspondence: G. Delsol, Department of Anatomical Pathology, CHU Purpan, 31059 Toulouse, France.
Received 31 January 1991; and in revised form 5 April 1991.

in ethanol-based Bouin's (Dubosq-Brazil) fluid or Bouin-Holland, but a few were fixed in B5 or 10% formalin, to assess the fixative-dependence of the staining intensity. A prior trypsination step was used for paraffin sections. In the study of lymphoid tumours, negative controls were used by the omission of BNH9 and BNF13 MoAbs or by their replacement with a non-relevant MoAb such as KL1; an anticytokeratin antibody (Immunotech, Marseille, France). Detailed immunophenotype of malignant lymphomas in our files was known because of the use of a broad panel of MoAbs directed against B-cell, T-cell, macrophage and activation associated antigens, and has been the subject of previous reports (Al Saati *et al.*, 1984; Delsol *et al.*, 1988). Staining of erythrocytes and blood-vessel endothelium served as positive controls.

*Reactivities with activated peripheral blood lymphocytes, normal lymphoid tissues, and malignant lymphomas*

Peripheral blood mononuclear cells from five healthy volunteers were stimulated with 1% PHA (Difco Lab) for 3 days. PHA-stimulated cells were tested for reactivity with BNH9 and BNF13 MoAbs on day 0, 1, 2 and 3, using the alkaline phosphatase: anti-alkaline phosphatase (APAAP) technique (Cordell *et al.*, 1984). In order to know whether antigens detected by these antibodies were of the activation type inducible by EBV (Calender *et al.*, 1987), five EBV-positive lymphoblastoid cell lines from own laboratory were tested by staining with the APAAP technique.

The reactivities of BNH9 and BNF13 MoAbs were investigated on normal tissues as well as reactive lymph nodes ($n = 20$) and tonsils ($n = 10$). In the initial study of the reactivity of these antibodies with malignant lymphomas, we noted an unexpected reactivity of some cases of ALC lymphomas (Ki-1 lymphomas) described by Stein *et al.* (1985; Delsol *et al.*, 1988). This finding prompted us to systematically investigate a large series of ALC lymphomas ($n = 127$) as well as NHL of non-anaplastic type ($n = 208$) and cases of HD of all subtypes ($n = 145$).

*Reactivities with non-lymphoid normal tissues and neoplasms*

A variety of surgically excised non-lymphoid human tumours from our tissue bank ($n = 153$) were tested. The reactivities with normal tissues were assessed on paraffin sections using the 'sausage' tissue block method (Battifora, 1986).

### Results

*Adsorption with synthetic oligosaccharides*

The results of haemagglutination-inhibition with synthetic oligosaccharides and tissue reactivities showed that BNF13 and BNH9 have H-related specificity, but react with different epitopes of the H-antigen. BNF13 was totally adsorbed by H type 2 suggesting binding with an epitope containing the trisaccharide alphaFuc(1-2)BetaGal(1-4)BetaGlcNAc and dependant upon the Beta(1-4)galactosyl linkage. The reactivity of BNH9 MoAb suggested a binding with an epitope containing either the alphaFuc(1-2)BetaGal(1-4)BetaGlcNac or alphaFüc(1-2)BetaGal(1-3)BetaGlcNAc trisaccharide present respectively on H and Y type 2 and H type 1. However, the adsorption by H type 4 could indicate that the reactivity of BNH9 MoAb was dependant on the disaccharide component alphaFuc(1-2)BetaGal present on H type 1, H type 2, H type 4 and Y type 2. These findings were further confirmed by the complete absence of immunostaining of epithelium and blood vessels when absorbed supernatants were tested for their activity on normal human tonsils.

*Reactivities with PHA-stimulated lymphocytes and Epstein-Barr Virus (EBV) + lymphoblastoid cell lines*

Many PHA-transformed lymphocytes showed an extremely weak, diffuse cytoplasmic staining with BNH9 and BNF13 antibodies by the sensitive APAAP procedure. In addition, except for one patient, a small percentage of cells showing a clear membrane staining with both antibodies, emerged on day 3.

All but one EBV+ lymphoblastoid cell lines showed 1–3% of cells positive for BNH9 and BNF13 MoAbs. The staining was strong and mainly membrane associated (Figure 1). These cell lines were of polyclonal B-lymphocytes expressing several activation antigens including the Ki-1 (CD30) antigen and the recently described CDw70 antigen (Al Saati *et al.*, 1989*a*).

*Reactivities with normal hematopoietic tissues*

Immunoperoxidase staining on frozen and paraffin sections gave similar results. On reactive lymph nodes, endothelial cells including post-capillary venules, reacted strongly with both antibodies (Figure 2). In addition, stellate reticulum cells of the marginal sinus were also strongly positive for the two MoAbs. By contrast, histiocytes, plasma cells and lymphoid cells including germinal centre cells and immunoblasts in the paracortical area, were consistently negative. In only two reactive lymph nodes, scarce positive small lymphocyte-like cells were found. Erythroblasts were considered as possible candidates for this positive reactivity. In normal spleen, only endothelial cells and sinus lining cells were found to be strongly positive. Variation of fixatives did not make a significant difference in the staining patterns. Staining in several randomly chosen bone marrow biopsy specimens was confined to endothelial cells, erythroblasts and a variable number of megakaryocytes. Myeloid cells were unequivocally negative.

**Figure 1** Reactivity of BNH9 antibody with Epstein-Barr virus positive lymphoblastoid cell line. Clear and strong membrane staining of two cells. APAAP staining technique × 800.

**Figure 2** BNH9 antibody staining of a fixed and paraffin-embedded reactive lymph node. Strong staining of endothelial cells (including postcapillary venules). Note absence of staining of both germinal centre and mantle zone lymphocytes. Immunoperoxidase with nuclear counterstain × 312.

*Reactivities with malignant lymphomas and related disorders*

Because of the fixation-resistant property of the antigens, only paraffin sections were used in the extended evaluation. However, in the early stages, BNH9 antibody consistently stained more numerous malignant cells than BNF13. Moreover, ten cases which were positive for BNH9 MoAb showed no positive cells with BNF13, while the opposite staining pattern, i.e. BNH9−/BNF13+, was not observed in any case. Thus, the study was mainly focused on the reactivity of BNH9 with paraffin embedded tissue sections from patients with lymphomas and related disease and the results are shown in Tables I, II and III.

*Anaplastic large cell (ALC) lymphomas (Tables I and II)* Tissues in 65 of the 127 cases (51%) reacted with BNH9; while only 25% of these tumours were also positive for BNF13. In a previous report (Delsol *et al.*, 1988), we had subdivided ALC lymphomas on the basis of the co-expression of Ki-1 (CD30) and epithelial membrane antigens (EMA) into three types: Ki-1 + /EMA + ; Ki-1 + /EMA − and Ki-1 − /EMA + . In the present study, 61/108 cases (56.5%) of Ki-1 + /EMA + ALC lymphomas were found to be positive for BNH9. Among tumours expressing either the Ki-1 antigen (nine cases) or EMA (ten cases) only one (11%) and three (30%) cases respectively, were positive for BNH9. The number of positive cells varied greatly from case to case but in the majority (40/65) of BNH9 positive cases, 50% to 100% of malignant cells were labelled. There were three cases in which BNH9 stained only 1% of malignant cells. Whatever the number of positive cells, the staining was intense, on the cell membranes, and commonly associated with a dot-like pattern in the paranuclear Golgi area (Figure 3). No other cells (except the positive control endothelial cells), were stained in BNH9 positive tumours. In addition, it was to noticed that, BNH9 positive ALCs were mainly of either T-cell origin (59%) or null phenotype (31%) (Table I). A significant difference was noted in the reactivities of BNH9 with ALC lymphomas in children as compared to those in adults. In children 29/41 (70.7%) tumours were positive for BNH9 as compared to 36/86 (41.8%) in adults ($P = 0.0023$). The observations need further detailed assessment.

The RBC phenotype was known in 46 patients. No significant differences were found between RBC phenotype, the secretor status and the reactivity with BNH9.

*NHL other than ALC (Table III)* Only 12 of 208 (5.7%) NHL were positive for BNH9. In these cases, the staining was intense and membrane associated similar to that of ALC lymphomas. Noteworthy was the finding that 11/12 these positive cases of NHL, were high grade lymphomas by mor-

**Table I** Anaplastic large cell lymphomas. Correlations of reactivity of BNH9 with phenotype

| Phenotype | Type 1 (BNH9 + /tested) | Type 2 (BNH9 + /tested) | Type 3 (BNH9 + /tested) |
|---|---|---|---|
| T-cell | 13/29 | 0/2 | ND |
| B-cell | 1/5 | ND | 0/2 |
| B + T | 1/2 | 0/2 | 0/2 |
| Null | 7/12 | ND | ND |
| Undetermined* | 39/60 | 1/5 | 3/6 |
| Total | 61/108 (56.5%) | 1/9 (11.1%) | 3/10 (30%) |

Types of anaplastic large cell lymphomas as previously reported (ref. Delsol *et al.*, 1988): Type 1: Ki-1 + , EMA + ; Type 2: Ki-1 + , EMA − ; Type 3: Ki-1 − , EMA + . − Ki-1 = CD30. − EMA = epithelial membrane antigen; ND: not done; *Undetermined because of non availability of frozen tissue. These cases were negative with anti-T, anti-B, and anti-macrophage antibodies reactive on fixed and paraffin-embedded specimens.

**Table II** BNH9 monoclonal antibody reactivity with malignant lymphomas and Hodgkin's disease

| | | No of cases | BNH9 + |
|---|---|---|---|
| Anaplastic large cell (ALC) lymphomas | | 127 | 65 (51%) |
| Non-Hodgkin's lymphomas other than ALC | | 208 | 12 (5.7%) |
| Hodgkin's disease | | 145 | |
| lymphocyte predominance | (LP) | 19 | 4 (21%) |
| nodular sclerosis | (NS) | 49 | 1 |
| mixed cellularity | (MC) | 66 | 1 |
| lymphocyte depletion | (LD) | 2 | 1 |
| unclassified | | 9 | 3 |

Details of the reactivities of non-Hodgkin's lymphomas other than ALC are given in Table III. Excluding LP subtype of Hodgkin's disease (see Chittal *et al.*, 1990 for reasons of exclusion), some classic Reed-Sternberg cells were positively stained in 6/126 (<5%) cases.

**Table III** Reactivities of non-Hodgkin's lymphomas (other than anaplastic large cell type) with BNH9 monoclonal antibody

| Type (Kiel classification) | BNH9 + / No. tested |
|---|---|
| *B-cell lymphomas* | |
| Low grade | |
| chronic lymphocytic leukaemia | 1/8 |
| hairy cell leukaemia | 0/7 |
| lymphoplasma-cytic/cytoid | 0/4 |
| centroblastic-centrocytic | 0/25 |
| centrocytic | 0/7 |
| other types | 0/12 |
| High grade | |
| centroblastic | 2/37 |
| immunoblastic | 2/19 |
| lymphoblastic | 1/9 |
| unclassified | 2/20 |
| *T-cell lymphomas* | |
| Low grade | 0/1 |
| chronic lymphocytic leukaemia | 0/1 |
| mycosis fungoides | 0/1 |
| Lennert's type | 0/3 |
| angioimmunoblastic type | 0/4 |
| T zone type | 0/3 |
| unclassified | |
| High grade | 1/15 |
| pleomorphic medium/large cell | 0/2 |
| immunoblastic | 0/1 |
| lymphoblastic | 1/3 |
| unclassified | |
| *Uncertain (B/T) high grade* | 1/7 |
| *Miscellaneous* | |
| not phenotyped | 0/9 |
| angioimmunoblastic lymphadenopathy | 0/2 |
| multiple myeloma* | 1/5 |
| true histiocytic tumours | 0/2 |
| acute lymphoblastic leukaemia | 0/1 |

*Positive case consisted of immature plasmablasts. N.B., 11/12 BNH9 positive cases of non-Hodgkin's lymphomas (other than ALC) were morphologically high grade type.

**Figure 3** A case of anaplastic large cell lymphoma stained with BNH9 antibody. Virtually all large cells show crisp membrane labelling. Strong staining of endothelial cells serves as positive control × 312. Inset: paranuclear dot-like reaction product in association with membrane staining at a higher magnification. Immunoperoxidase with nuclear counterstain × 800.

phologic criteria. True 'malignant histiocytosis' and histiocytosis X did not react with BNH9.

The RBC phenotype was known in 32 patients in NHL. Similar to ALC lymphomas, no significant differences were found between their RBC phenotype, secretor status and the reactivity with BNH9.

*Hodgkin's disease (HD) (Table II)* In four of the 19 cases of lymphocyte predominance HD, some lympho-histiocytic cells (L and H type) reacted with BNH9 antibody. Interestingly, one of these four cases, reported in a separate publication, transformed into high grade large B-cell lymphoma, the cells of which were also positive for BNH9 (Chittal *et al.*, 1990). No staining on Reed-Sternberg cells and variants was found in 120 cases; only endothelial cells (used as positive controls), were stained (Figure 4). In only 6/126 (4.7%) cases of the other histological types of Hodgkin's disease (nodular sclerosis, mixed cellularity, lymphocyte depletion and unclassified: total = 126 cases), rare morphologically typical Reed-Sternberg cells were found to be positive for BNH9.

*Reactivities with non-lymphoid normal tissues and tumours*

A large panel of normal human tissues obtained from paraffin embedded biopsy specimens (normal multi-tissue block) were stained to specify the reactivities of BNH9 and BNF13. In all organs, endothelial cells were strongly labelled with the two antibodies. Minor differences were found in the reactivities of BNH9 and BNF13 on cells other than endothelial cells. Squamous cells of the skin, tonsil and oesophagus were positive for both antibodies. Sebaceous glands were labelled, in addition to epidermal cells and sweat glands, but only with BNF13. Varying proportions of glandular structures of the bronchus, gastrointestinal tract, salivary glands and pancreas (except for islets cells) were also stained. A small proportion of tubules in kidney were labelled. Except for the usual reactivity with blood vessels, no significant staining was observed in liver, heart, brain, testis and ovary.

Reactivities of BNH9 and BNF13 antibodies with non-lymphoid tumours were assessed on frozen and paraffin sections using the multi-tumour (sausage) tissue block method. Among 153 tested 76 were positive for BNH9. Major positive reactivities were found with carcinomas of the lung (squamous cell: 9/9; adenocarcinoma: 8/9; small cell carcinoma: 3/12), thyroid (7/12), gastrointestinal tract (eosophagus: 1/2; gastric: 6/8; colon: 3/9), pancreas (1/2), liver (1/4), breast (10/11), kidney (1/7), urinary bladder (4/5), prostate (3/5) and uterine cervix (2/2). Soft tissue tumours were negative for BNH9 with the exception of angiosarcoma and synovial sarcoma. Glial tumours and meningiomas were negative.

**Figure 4** Staining of a case of Hodgkin's disease with BNH9 antibody in which a classic Reed-Sternberg cell (short arrow) is negative whereas strong staining of endothelial cells is positive control. Immunoperoxidase with nuclear counterstain × 312.

### Discussion

The results suggest that BNH9 and BNF13 are 'anti-H' antibodies but react with different epitopes of the H-antigen. BNH9 antibody reacted with H type 1, H type 2, H type 4 and Y (type 2) (Oriol *et al.*, 1987). The lack of adsorption by H type 3 synsorbs is difficult to explain. BNF13 antibody was adsorbed only by H type 2 and Y type 2 and type 2 precursor, identical to previously obtained results (Oriol *et al.*, 1987). The findings obtained so far, are relevant to the reactivities of these antibodies on paraffin sections, because carbohydrates rich glycoproteins are usually resistant to various fixatives (Stross *et al.*, 1989).

As expected, these two antibodies reacted with erythrocytes and endothelial cells, since H antigens are expressed on these cells regardless of the secretor status (Kimmel *et al.*, 1986; Le Pendu *et al.*, 1989). Similarly, the reactivity of many normal epithelial cells as well as many carcinoma cells of diverse origin, is in agreement with previous studies on the tissue distribution of H and Y antigens (Feizi, 1985; Coon & Weinstein, 1986; Vowden *et al.*, 1986).

Lymphocytes are known to express ABH and Lewis determinants under the control of the Se and Le genes (Dunstan, 1986; Oriol *et al.*, 1980). However, these antigens are absorbed from the plasma and appear to be primarily of H type 1 (Mollicone *et al.*, 1988; Oriol *et al.*, 1980). Lymphocytes were unreactive with BNH9 and BNF13 MoAbs by cytofluorometric analysis (data not shown). Lymphoid cells were also consistently negative in reactive lymph nodes. These antibodies reacted only with endothelial cells of blood vessels in all lymph nodes, but occasional stellate cells of the marginal sinus appeared to be positively stained. Rare cells with appearance of lymphocytes found in two cases, were likely erythroblasts.

Due to the acquisition of H and Y antigens by ALC lymphomas, which are known to arise from activated lymphoid cells, there was a high probability that these antigens could be promoted on PHA-stimulated blood lymphocytes. We did observe clear staining with BNH9 and BNF13 MoAbs not only with PHA-stimulated lymphocytes but also in a small percentage of cells from EBV + lymphoblastoid cell lines. This latter finding is in agreement with that of Mollicone and co-workers (1988) who stressed that the H gene product can indeed be expressed in lymphoblastoid cell lines. Changes of glycosylation pattern have been described in other virus infected cells such as Herpes virus infected cells (Ray & Blough, 1978) and it has also been recently reported that hapten Y could be expressed by lymphocytes infected with the human immunodeficiency (HIV) virus (Adachi *et al.*, 1988). However, we did not find staining of lymphocytes in reactive lymph nodes from known HIV-seropositive patients, with BNH9 and BNF13 MoAbs.

Although not expressed on normal lymphocytes, H determinant was previously found to be expressed in B acute lymphoblastic leukaemias (Koller *et al.*, 1987). Expression of H and/or Y antigens detected by BNH9 MoAb in over 50% of ALC lymphomas is in striking contrast to its rare occurrence in the other lymphoid neoplasms (5.7% of NHL in our cases). As previously demonstrated, the expression of blood group antigens in normal tissues may be dependent on the secretor status, some of these antigens being taken up from the serum, particularly on lymphocytes (Lloyd, 1987; Mollicone *et al.*, 1988; Oriol *et al.*, 1980). No significant correlation was found with BNH9 staining with either the secretor status or with the blood groups of our patients. In addition, passive absorption from serum was ruled out by the finding, that in the majority of positive cases, the membrane staining was associated with a dot-like staining in the Golgi area. This paranuclear staining pattern is consistent with the fact that glycoproteins and glycolipids are glycoslated in the Golgi apparatus (Alberts *et al.*, 1983). Lastly, the wide disparity between BNH9 staining in ALC lymphomas and other lymphoid neoplasms, makes the passive absorption phenomenon unlikely. Thus, expression of H and Y antigens may represent biological distinctiveness of ALC lymphomas among

NHL. Since these antigens were inducible by stimulation of lymphocytes by mitogens such as PHA or by EBV infection, they are candidates as activation antigens, like other activation markers such as the Ki-1 (CD30) antigen (Stein *et al.*, 1985) and the CDw70 antigen (Al Saati *et al.*, 1989*a*). However, the reactivity with BNH9 antibody suggests that ALC lymphomas occurring in adults are more heterogeneous than similar tumours in children (41.8 *vs* 70.7% were respectively positive for BNH9).

In non-lymphoid tumours e.g. urothelial carcinomas, dramatic changes in the expression of blood group antigens such as the loss of ABH and $Le^a$ antigens, are usually associated with a more aggressive potential, compared to those in which these antigens are preserved (Coon & Weinstein, 1986; Orntoft *et al.*, 1989). Preliminary results using univariate analysis suggest that adults with BNH9 positive ALC lymphomas fare worse in comparison to those with BNH9 negative tumours. These findings need to be confirmed by multivariate analysis and controlled prospective studies. No such differences was found in children.

A possible diagnostic use of BNH9 antibody, is in making the distinction between neoplastic cell-rich HD and ALC lymphomas; a problem expected to arise in approximately 10% of cases in our experience. As found in this study, ALC lymphomas, more often than not, show CD30 +, EMA +, BNH9 + phenotype whereas typical Reed-Sternberg cells in HD most frequently co-express CD15 and CD30 antigens (Chittal *et al.*, 1988). EMA is not expressed by classic Reed-Sternberg cells (Chittal *et al.*, 1988). Similarly, we found the antigens detected by BNH9 antibody to be expressed rarely by Reed-Sternberg cells in HD (Table II). Even though the differential expression of CD15 (X-hapten) could further assist in the separation of these entities, caution needs to be exercised, as up to 22% of cases of ALC lymphomas may express the CD15 antigen (Delsol *et al.*, 1988). The frequent expression of EMA and H and Y antigens by ALC lymphomas indicates that HD and ALC lymphomas may not be as closely related entities as previously suggested on the basis of CD30 expression (Stein *et al.*, 1985).

The mechanism responsible for expression of H and Y antigens by ALC lymphomas remains to be clarified, but there may be possible explanations. As discussed above, a viral infection could be involved in abnormal glucosyltransferase activity. Alternatively, an abnormality of glycosyltransferase activity may be the result of a specific chromosomal abnormality found in ALC lymphomas. Mason *et al.* (1990) have demonstrated that ALC lymphomas frequently show a translocation involving exchange of material between chromosomes 2 and 5 at bands 2p23 and 5q35. We found the same translocation in two BNH9-positive ALC lymphomas of our series in which cytogenetic analysis was performed (data not shown). Much further investigation will be needed to clarify this important issue.

This work was supported by grants from Association pour la Recherche sur le Cancer and Fondation pour la Recherche Médicale. We greatly appreciate the help of Professor R. Oriol, CNRS ER-281, Paris, France, in the characterisation of the antibodies using Chembiomed oligosaccharides. SMC (Memorial University Medical School, St. John's, NF, Canada) was supported by the French Ministry of Education.

### References

ADACHI, M., HAYAMI, M., KASHIWAGI, N. & 7 others (1988). Expression of $Le^y$ antigen in human immunodeficiency virus-infected human T cell lines and in peripheral lymphocytes of patients with acquired immune deficiency syndrome (AIDS) and AIDS-related complex (ARC). *J. Exp. Med.*, **167**, 323.

ALBERTS, B., BRAY, D., LEWIS, J., RAFF, M., ROBERTS, K. & WATSON, J.D. (1983). (eds), *Molecular Biology of the Cell.* Garland Publishing Inc., New York and London, p. 319.

AL SAATI, T., LAURENT, G., RIGAL-HUGUET, F., CAVERIVIERE, P. & DELSOL, G. (1984). Reactivity of Leu 1 and T 101 monoclonal antibodies with B cell lymphomas (correlation with other immunological markers). *Clin. Exp. Immunol.*, **58**, 631.

AL SAATI, T., BLANCHER, A., CALVAS, P., NEULAT-DUGA, I. & DELSOL, G. (1987). Production of monoclonal antibodies using spleen cells from nude mice bearing human tumors. *Ann. Pathol.*, **7**, 1.

AL SAATI, T., MAZEROLLES, C., CASPAR, S. & 8 others (1989*a*). Production of two monoclonal antibodies identifying a novel activation antigen using spleen cells from nude mice bearing HLY1 cell line. In *Leucocyte Typing IV*, Knapp, W. *et al.* (eds). Oxford University Press, Oxford, p. 452.

AL SAATI, T., CASPAR, S., BROUSSET, P. & 8 others (1989*b*). Production of anti-B monoclonal antibodies (DBB.42, DBA.44, DNA.7 and DND.53) reactive on paraffin-embedded tissues with a new B-lymphoma cell line grafted into athymic nude mice. *Blood*, **74**, 2476.

BATTIFORA, H. (1986). The multitumor (sausage) tissue block. Novel method for immunohistochemical testing. *Lab. Invest.*, **55**, 244.

BLANCHER, A., AL SAATI, T., MARTY, Y. & DELSOL, G. (1988). Mouse monoclonal antibodies against the H and I blood group determinant, produced by hybridomas from lymphocytes of athymic nude mice. In *Monoclonal Antibodies Against Human Red Blood Cell and Related Antigens.* Rouger, Ph. & Salmon, Ch. (eds), Paris: Librairie Arnette, p. 220.

CALENDER, A., BILLAUD, M., AUBRY, J.P., BANCHEREAU, J., VUILLAUME, M. & LENOIR, G.M. (1987). Epstein-Barr virus (EBV) induces expression of B-cell activation markers on in vitro infection of EBV-negative B-lymphoma cells. *Proc. Natl Acad. Sci. USA*, **84**, 8060.

CHITTAL, S.M., CAVERIVIERE, P., SCHWARTING, R. & 5 others (1988). Monoclonal antibodies in the diagnosis of Hodgkin's disease. The search for a rational panel. *Am. J. Surg. Pathol.*, **12**, 9.

CHITTAL, S.M., ALARD, C., ROSSI, J.F. & 4 others (1990). Further phenotypic evidence that nodular, lymphocyte predominance Hodgkin's disease is a large B-cell lymphoma in evoluation. *Am. J. Surg. Pathol.*, **14**, 1024.

COON, J.S. & WEINSTEIN, R.S. (1986). Blood group-related antigens as markers of malignant potential and heterogeneity in human carcinomas. *Hum. Pathol.*, **17**, 1089.

CORDELL, J., FALINI, B., ERBER, O.N. & 6 others (1984). Immunoenzymatic labelling of monoclonal antibodies using immune complexes of alkaline phosphate and monoclonal anti-alkaline phosphatase (APAAP Complexes). *J. Histochem. Cytochem.*, **31**, 219.

DELSOL, G., AL SAATI, T., GATTER, K.C. & 7 others (1988). Coexpression of epithelial membrane antigen (EMA), Ki-1, and interleukin-2 receptor by anaplastic large cell lymphomas. Diagnostic value in so-called malignant histocytosis. *Am. J. Pathol.*, **130**, 59.

DUNSTAN, R.A. (1986). Status of major red cell blood group antigens on neutrophils, lymphocytes and monocytes. *Br. J. Haematol.*, **62**, 301.

FEIZI, T. (1985). Demonstration by monoclonal antibodies that carbohydrate structures of glycoproteins and glycolipids are oncodevelopmental antigens. *Nature*, **314**, 53.

HAKOMORI, S.I., KANNAGI, R. (1983). Glycospingolipids as tumor-associated and differentiation markers. *J. Natl Cancer Inst.*, **71**, 231.

KIMMEL, K.A., CAREY, T.E., JUDD, W.J., MCCLATCHEY, K.D. (1986). Monoclonal antibody (G10) to a common antigen of human squamous cell carcinoma: binding of the antibody to the H type 2 blood group determinant. *J. Natl Cancer Inst.*, **76**, 9.

KOHLER, G. & MILSTEIN, C. (1976). Derivation of specific antibody producing tissue culture and tumor lines by cell fusion. *Europ. J. Immunol.*, **6**, 511.

KOLLER, U., BETTELHEIM, P., MAJDIC, O., LANDSDORP, P.M., ITETTEROO, P.A.T. & KNAPP, W. (1987). Expression of blood group H antigen on blast cells in leukemias and CML blast crises. In *Genotypic, Phenotypic and Functional Aspects of Haematopoiesis.* Grignani, F., Martelli, M.F. & Mason, D.Y. (eds). Serono Symposia, Raven Press NY, 41, p. 109.

LEMIEUX, R.U. (1978). Human blood groups carbohydrate chemistry. *Chem. Soc. Rev.*, **7**, 423.

LE PENDU, J., DALIX, A.M., MOLLICONE, R., CRAINIC, K. & ORIOL, R. (1989). Expression of ABH, Lewis and related tissue antigens in the human thymus. *J. Immunogenetics*, **16**, 19.

LLOYD, K.O. (1987). Blood group antigens as markers for normal differentiation and malignant change in human tissues. *Am. J. Pathol.*, **87**, 129.

MASON, D.Y., BASTARD, C., RIMOKH, R. & 9 others (1990). CD30-positive large cell lymphoma ('Ki-1 lymphoma') are associated with a chromosomal translocation involving 5q35. *Br. J. Haematol.*, **74**, 161.

MOLLICONE, R., CAILLARD, T., LE PENDU, J. & 4 others (1988). Expression of ABH and X ($Le^x$) antigens on platelets and lymphocytes. *Blood*, **71**, 1113.

ORIOL, R., DANILOVS, J., LEMIEUX, R., TERASAKI, P. & BERNOCO, D. (1980). Lymphocytotoxic definition of combined ABH and Lewis antigens and their transfer from sera to lymphocytes. *Hum. Immunol.*, **3**, 195.

ORIOL, R., GANE, P., ROUGER, Ph. & MOLLICONE, R. (1987). Inhibition of haemagglutination with synsorbs and salivas by monoclonal antibodies against non-A, non-B glycuroconjugates. In *First International Workshop On Monoclonal Antibodies Against Human Red Blood Cell and Related Antigens*. Rouger, Ph, Anstee, D. & Salmon, Ch. (eds), Paris, Librairie Arnette, p. 671.

ORNTOFT, T.F., WOLF, H., CLAUSEN, H., DABELSTEEN, E. & HAKOMORI, S.I. (1989). Blood group ABH-related antigens in normal and malignant bladder urothelium: possible structural basis for the deletion of type-2 chain ABH antigens in invasive carcinomas. *Int. J. Cancer*, **43**, 774.

RAY, E.K. & BLOUGH, H.A. (1978). The effect of herpes virus infection and 2-deoxy-D-glucose on glycosphingolipids in BHK-21 cells. *Virology*, **88**, 118.

SCHOENTAG, R., PRIMUS, F.J. & KUHNS, W. (1987). ABH and Lewis blood group expression in colon carcinoma. *Cancer Res.*, **47**, 1695.

STEIN, H., MASON, D.Y., GERDES, J. & 8 others (1985). The expression of Hodgkin's disease associated antigen Ki-1 in reactive and neoplastic lymphoid tissue: evidence that Reed-Sternberg cells and histocytic malignancies are derived from activated lymphoid cells. *Blood*, **66**, 848.

STROSS, W.P., WARNKE, R.A., FLAVELL, D.J. & 4 others (1989). Molecule detected in formalin fixed tissue by antibodies MT1, DFT1, and L60 (Leu-22) corresponds to CD43 antigen. *J. Clin. Pathol.*, **42**, 953.

SZULMAN, A.E. (1962). The histological distribution of the blood group substances in man as disclosed by immunofluorescence. II. The H antigen and its relation to A and B antigens. *J. Exp. Med.*, **115**, 977.

VOWDEN, P., LOWE, A.D., LENNOX, E.S. & BLEEHEN, N.M. (1986). The expression of ABH and Y blood group antigens in benign and malignant breast tissue: the preservation of the H and Y antigens in malignant epithelium. *Br. J. Cancer*, **53**, 313.